# Identification of new target proteins of a Urotensin-II receptor antagonist using transcriptome-based drug repositioning approach

**DOI:** 10.1038/s41598-021-96612-0

**Published:** 2021-08-24

**Authors:** Gyutae Lim, Chae Jo Lim, Jeong Hyun Lee, Byung Ho Lee, Jae Yong Ryu, Kwang-Seok Oh

**Affiliations:** 1 Data Convergence Drug Research Center, Korea Research Institute of Chemical Technology, 141 Gajeong-ro, Yuseong-gu, Daejeon, 34114 Republic of Korea; 2 Department of Medicinal and Pharmaceutical Chemistry, University of Science and Technology, 217 Gajeong-ro, Yuseong,-gu, Daejeon, 34113 Republic of Korea; 3 Department of Biotechnology, Duksung Women’s University, 33 Samyang-ro 144-gil, Dobong-gu, Seoul, 01369 Republic of Korea

**Keywords:** Computational models, Gene ontology, Virtual drug screening

## Abstract

Drug repositioning research using transcriptome data has recently attracted attention. In this study, we attempted to identify new target proteins of the urotensin-II receptor antagonist, KR-37524 (4-(3-bromo-4-(piperidin-4-yloxy)benzyl)-*N*-(3-(dimethylamino)phenyl)piperazine-1-carboxamide dihydrochloride), using a transcriptome-based drug repositioning approach. To do this, we obtained KR-37524-induced gene expression profile changes in four cell lines (A375, A549, MCF7, and PC3), and compared them with the approved drug-induced gene expression profile changes available in the LINCS L1000 database to identify approved drugs with similar gene expression profile changes. Here, the similarity between the two gene expression profile changes was calculated using the connectivity score. We then selected proteins that are known targets of the top three approved drugs with the highest connectivity score in each cell line (12 drugs in total) as potential targets of KR-37524. Seven potential target proteins were experimentally confirmed using an in vitro binding assay. Through this analysis, we identified that neurologically regulated serotonin transporter proteins are new target proteins of KR-37524. These results indicate that the transcriptome-based drug repositioning approach can be used to identify new target proteins of a given compound, and we provide a standalone software developed in this study that will serve as a useful tool for drug repositioning.

## Introduction

Novel drug development is still a time-consuming and expensive task^1,2^ because it is difficult to predict side effects or toxicity in advance^3^. Even if a drug is successfully developed and approved, its frequency of use decreases over time because of the emergence of more efficient drugs and the occurrence of unexpected resistance^4^. However, since approved drugs have already passed the verification of in vivo toxicity and side effects through clinical trials, drug repositioning or repurposing approaches can find potential new target proteins and therefore improve drug usability^5,6^. This is more efficient and easier than the development of an entirely new drug^7,8^. For this reason, the drug repositioning approach has recently been favored for new drug development.

Computational drug repositioning is an efficient approach for screening new target proteins. Various computational drug repositioning methods have been developed using transcriptome data to identify potential new target proteins for drugs, such as comparing gene expression profile changes between disease models and drug treatment conditions^9,10^, prediction of drug-protein interactions^11–13^, and network integration^14,15^. In addition, the Connectivity Map (CMap) database has provided a total of 564 gene expression profiles of 143 distinct bioactive small-molecule perturbagens representing 453 individual instances since 2006^16^. Many computational methods use CMap to predict unknown drug-disease associations based on the inverse correlation of gene expression patterns^17,18^. The Library of Integrated Network-based Cellular Signatures (LINCS) L1000 database extends CMap to include a larger number of gene expression profiles^19^. The LINCS L1000 database provides gene expression profiles induced by approximately 33,000 small molecules in 99 cell lines, and is therefore highly useful for new drug discovery and repositioning^20,21^.

We previously developed novel urotensin-II receptor antagonists such as KR-36676^22^ and KR-36996^23,24^, which are derived from benzo[*b*]thiophene-2-carboxamide. These compounds acted selectively against the urotensin-II receptor with good affinity (Ki = 0.7 nM and 4.44 nM for KR-36676 and KR-36996, respectively) in cellular events such as stress fiber formation and cellular hypertrophy. However, drug development of these compounds has since been discontinued. This decision was made after preliminary efficacy data from a proof-of-concept program yielded unsatisfactory results and identified cardiotoxicity issues. In contrast, KR-37524, (4-(3-bromo-4-(piperidin-4-yloxy)benzyl)-*N*-(3-(dimethylamino) phenyl) piperazine-1-carboxamide dihydrochloride), which was developed in tandem with KR-36676 and KR-36996, was found to be safe for toxicity issues, including cardiotoxicity, although affinity (Ki = 37 nM) was relatively low compared to that of other drugs^25^. In addition, KR-37524 is an analogue of the piperazine-carboxamide family and these analogs exhibit various biological activities such as platelet-derived growth factor receptor (PDGFR) inhibitors^26^, monoacylglycerol lipase (MAGL) inhibitor^27^, serotonin (5-HT1B) receptor antagonists^28^, chemokine receptor (CCR) antagonists^29,30^ or fatty acid amide hydrolase (FAAH) inhibitors^31–33^. Therefore, we tried to identify new target proteins of KR-37524 instead of the urotensin-II receptor by taking advantage of the toxicity-safe KR-37524.

**Figure 1:**
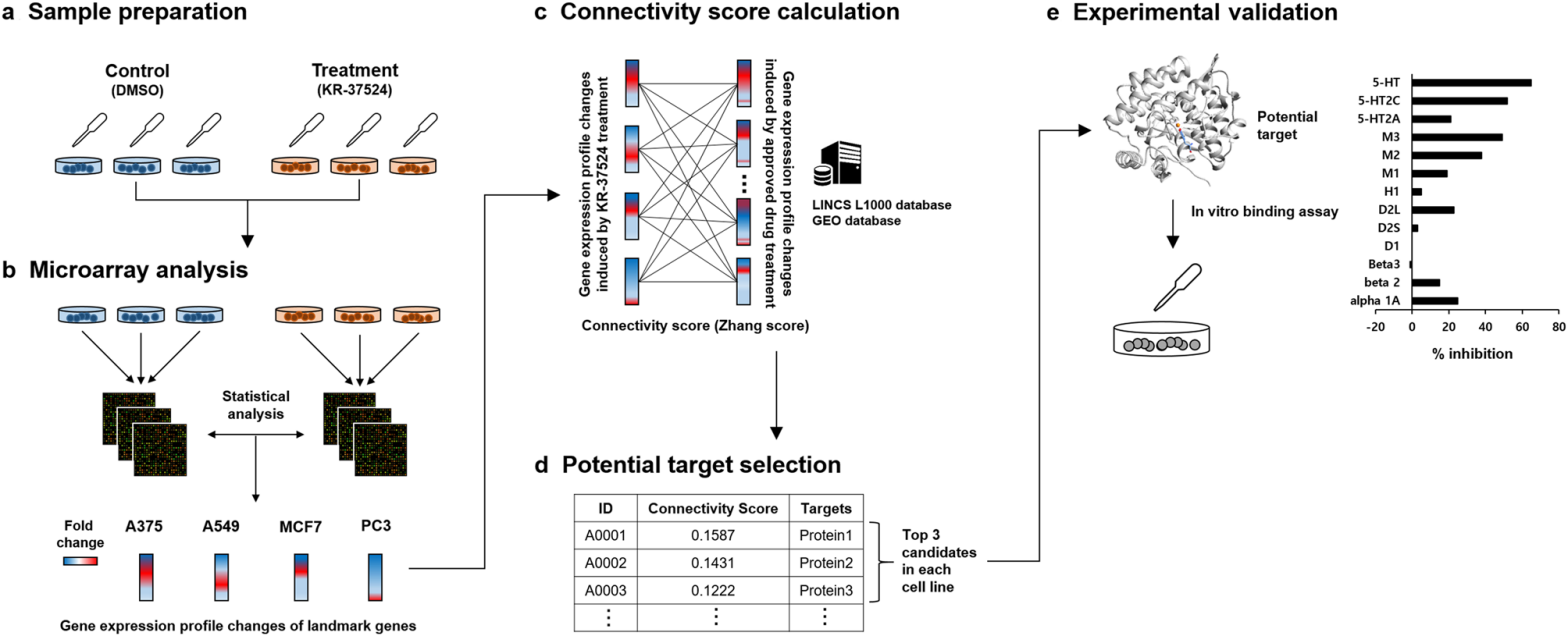
Overall scheme of this study. (**a**) Samples were prepared from four cell lines treated with KR-37524 or dimethyl sulfoxide (DMSO). (**b**) Microarray data of KR-37524 induced transcriptome changes were generated from this sample, and the fold change values were calculated to measure DEGs for the 978 landmark genes. (**c**) The gene expression patterns were compared with the LINCS L1000 data to find similar perturbagen signatures, and (**d**) the top three candidate target proteins were selected using the Zhang score for each cell line. (**e**) Finally, experimental validation was performed to confirm the affection of KR-37524 on the candidate target protein. The protein structure was drawn by Chimera software^34^ (www.cgl.ucsf.edu/chimera).

In this study, we identified the new target proteins of KR-37524 using a transcriptome-based drug repositioning approach to expand the new indication of KR-37524 (Fig. 1). To do this, we first generated the gene expression profile changes induced by KR-37524 in each of the four cell lines (A375, A549, MCF7, and PC3). We then compared the gene expression profile changes induced by KR-37524 in each cell line to the approved drug-induced gene expression profile changes provided in the LINCS L1000 database and found approved drugs with similar gene expression profile changes. Here, the similarity between the changes in the two gene expression profiles was calculated using a connectivity score. Specifically, we selected the top three approved drugs (12 drugs in total) with the highest connectivity score for each cell line, and selected their target proteins as potential target proteins of KR-37524. Among them, seven potential target proteins were experimentally validated through an in vitro binding assay, confirming that the neurologically regulated serotonin transporter protein is a new target for KR-37524. We also provide standalone software used in the current study to generalize our strategy (i.e., transcriptome-based drug repositioning).

## Results

### KR-37524-induced gene expression profile data analysis

First, we obtained gene expression profiles after treatment with DMSO (control group) and KR-37524 (treatment group) at 10 μM for 6 h by using microarray experiments in each of the four cell lines (i.e., A375, A549, MCF7, and PC3) (detailed in the Materials and Methods). Since the experiments were performed in triplicate, the mean value of gene expression for each gene was used. Then, we calculated the gene expression profile changes in each cell line by dividing the gene expression of the treatment group (KR-37524 treatment) by the gene expression of the control group (DMSO treatment). Thus, when the absolute value of the fold change (FC) was lower than 1, the expression lower than that of the control group was marked with a negative (−) sign.

**Figure 2:**
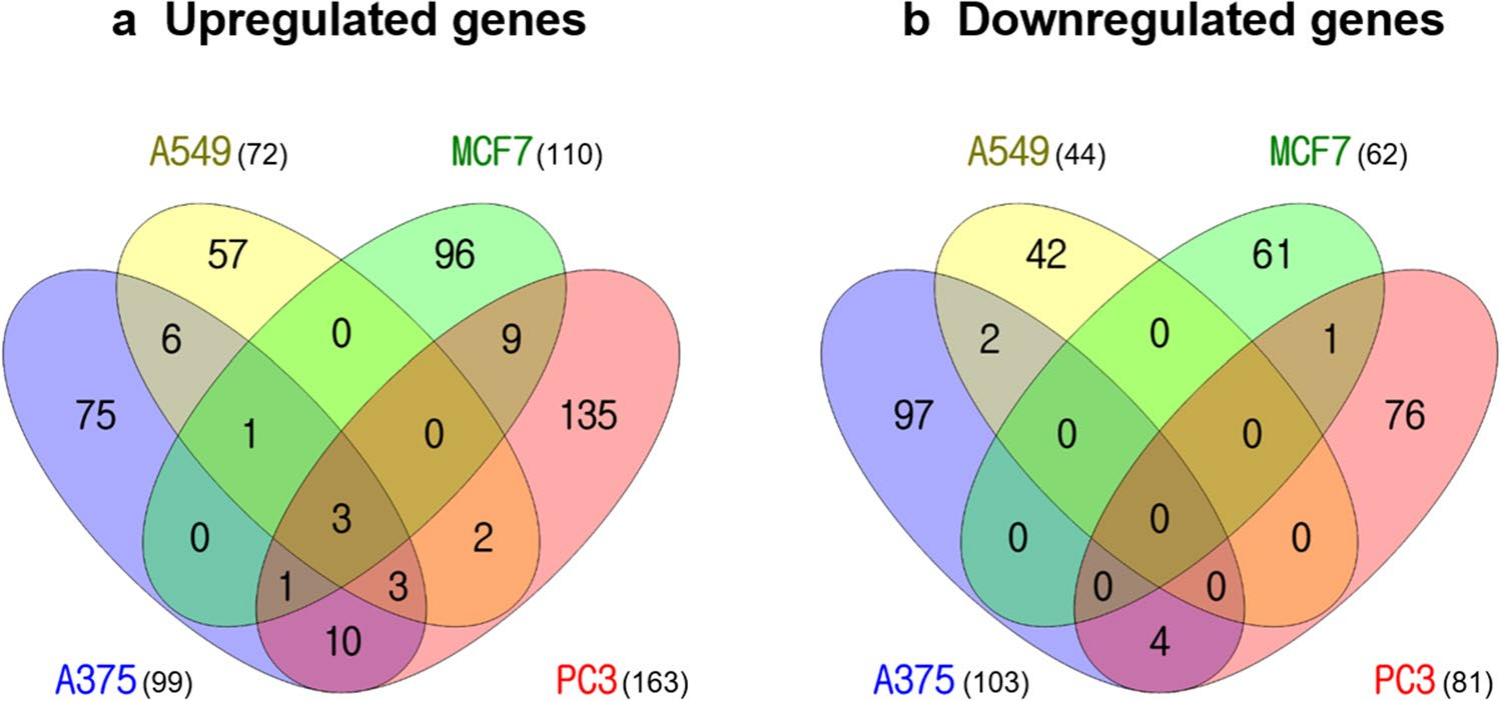
Venn diagram for the differentially expressed gene (DEG) lists in four cell lines. (**a**) Upregulated genes and (**b**) downregulated genes. The crossing areas show the commonly changed DEGs. Statistically significant DEGs were defined using *p* < 0.05 and |FC| $\ge$ 1.5 as cut-off. Venny website was used for drawing Venn diagrams (https://bioinfogp.cnb.csic.es/tools/venny/index.html).

Next, we analyzed the differentially expressed genes (DEGs) in each cell line (*p* < 0.05, |FC | $\ge$ 1.5). In A375 cells, there were 99 upregulated genes and 103 downregulated genes, and in A549 cells, there were 72 upregulated and 44 downregulated genes. In MCF7 cells, 110 upregulated genes and 62 downregulated genes were identified. Finally, in PC3 cells, there were 163 and 81 upregulated and downregulated genes, respectively. In all cell lines, there were a total of three commonly upregulated genes, while there were no commonly downregulated genes (Fig. 2 and Table S1). Commonly upregulated genes were *HMGCR* (3-hydroxy-3-methylglutaryl-CoA reductase), *LPIN1* (Lipin 1), and *SQLE* (Squalene epoxidase). *HMGCR* is the main target of statins, a class of cholesterol-lowering drugs, *LPIN1* controls fatty acid metabolism at different levels, and *SQLE* is considered to be a rate-limiting enzyme in steroid biosynthesis. All three genes are known to primarily regulate the synthesis and metabolism of lipids, such as cholesterol.

### GO and KEGG pathway enrichment analysis

**Figure 3:**
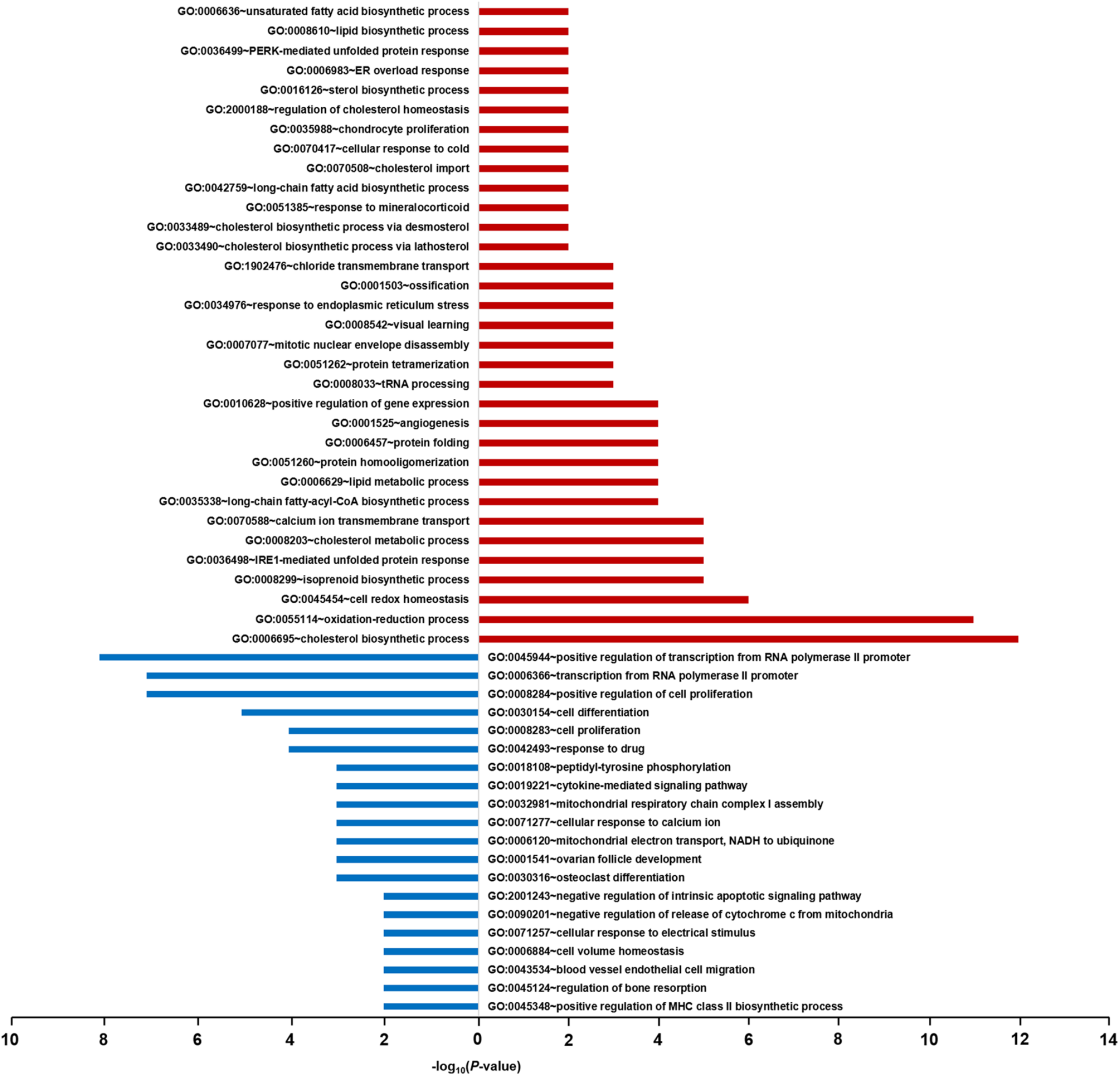
Results of GO enrichment analysis for Biological Process. The red and blue bars represent the results of GO enrichment analysis using upregulated genes and downregulated genes, respectively.

Gene ontology (GO) enrichment^35^ and Kyoto Encyclopedia of Genes and Genomes (KEGG) pathway^36^ enrichment analyses were performed for gene sets with |FC| $\ge$ 1.5 and *p* < 0.05 (i.e., DEGs) in each cell line (Tables S2 and S3). GO enrichment analysis was performed using the Database for Annotation, Visualization and Integrated Discovery (DAVID)^37,38^. In this study, we only considered the biological process (BP) sub-ontology among three sub-ontologies (cellular component, biological process, and molecular function). Significantly enriched BP GO terms (*p* < 0.05) were extracted from each cell line, and the genes from each cell line were merged to investigate the changes in the overall gene expression profile induced by KR-37524. A total of 54 upregulated and 36 downregulated genes were used for GO enrichment analysis, and the results are indicated in red and blue, respectively (Fig. 3). The top-ranked BP GO terms of upregulated genes were cholesterol biosynthetic process (22.2%), and the genes involved were *SQLE*, *IDI1*, *MVK*, *HMGCS1*, *INSIG1*, *MSMO1*, *DHCR24*, *HMGCR*, *DHCR7*, *HSD17B7*, *LSS*, and *FDFT1*. However, the overall number of downregulated genes was low, and the highest-ranked BP GO terms included the positive regulation of transcription from RNA polymerase II promoter (22.9%), transcription from RNA polymerase II promoter (20.0%), positive regulation of cell proliferation (20.0%), cell differentiation (14.3%), and cell proliferation (11.4%) (Table S4).

**Figure 4:**
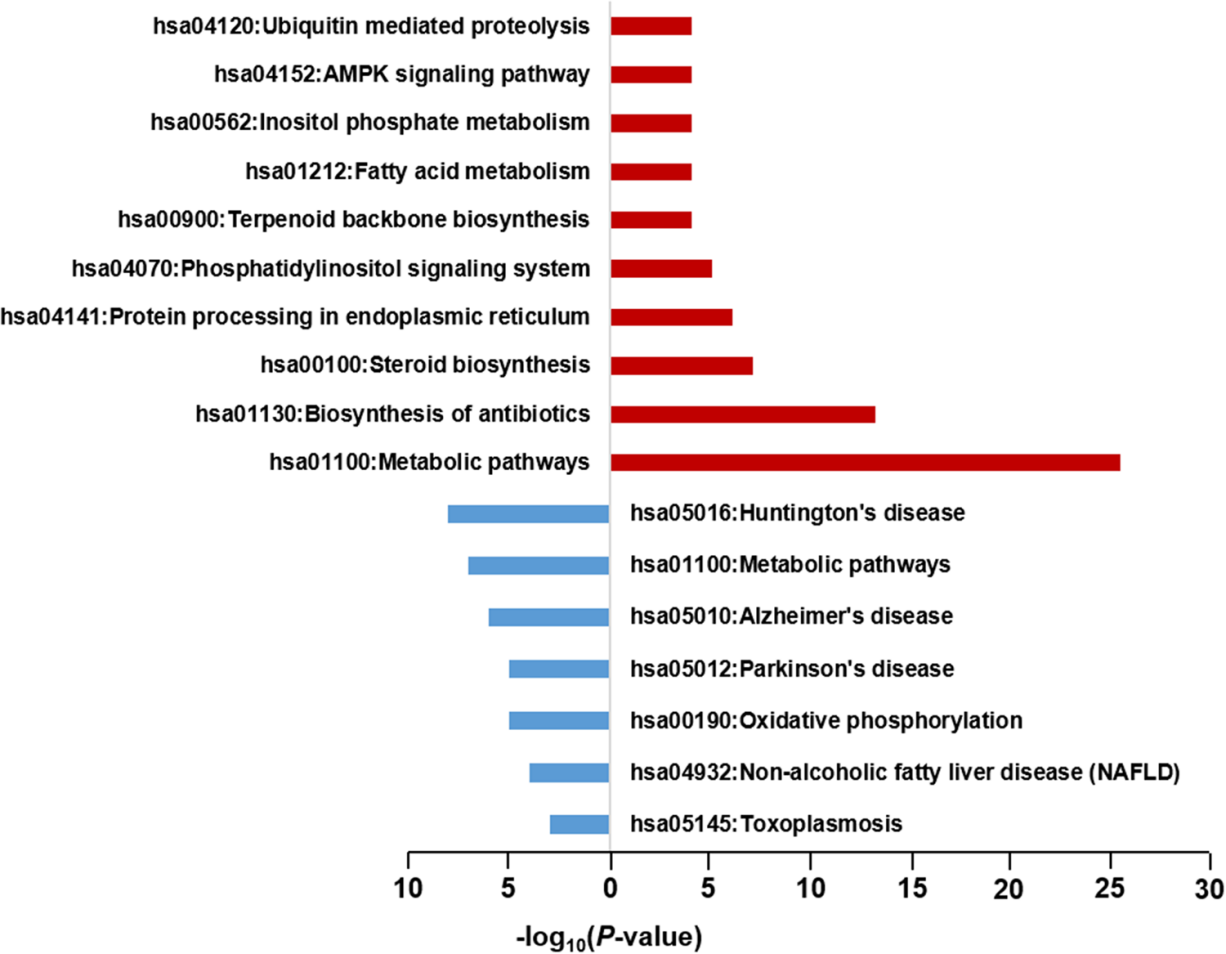
Results of KEGG pathway enrichment analysis. The red and blue bars represent the results of KEGG pathway analysis using upregulated genes and downregulated genes, respectively.

As a result of KEGG pathway enrichment analysis, the significantly enriched pathways of 40 upregulated and 13 downregulated genes were identified (*p* < 0.05) from the merged gene list of each cell line (Fig. 4). The top-ranked pathway for upregulated genes was the metabolic pathway (64.1%). In addition, biosynthesis of antibiotics (33.3%) and steroid biosynthesis pathway (17.9%) were highly ranked. Metabolic pathway genes included *PI4K2B*, *IDI1*, *MTMR3*, *MVK*, *MSMO1*, *HMGCR*, *HSD17B7*, *GK2*, *MTM1*, *FDFT1*, *AOC2*, *HMGCS1*, *IDH1*, *ACSL4*, *DHCR24*, *LSS*, *SQLE*, *GNPDA1*, *PSAT1*, *FASN*, *CYP1A2*, *DHCR7*, *LPIN1*, *BCAT1*, and *LDHAL6B*. The top-ranked pathway for downregulated genes was Huntington's disease pathway (61.5%) and included *NDUFA13*, *NDUFS7*, *NDUFA1*, *PPIF*, *CLTB*, *POLR2F*, *UQCR11*, and *POLR2I* (Table S5).

### Selection of candidate target proteins using LINCS L1000 data search

To identify new target proteins of KR-37524, we compared the gene expression profile changes induced by KR-37524 with each perturbagen in the LINCS L1000 dataset. To compare the similarity, we calculated the connectivity score developed by Zhang^39^ between the gene expression profile changes of KR-37524 and each perturbagen in the LINCS L1000 database. In each cell line, the three drugs with the highest connectivity scores were selected (i.e., 12 drugs in total), and the LINCS perturbagen ID was mapped to the DrugBank ID list for comparison with approved drugs. The top three selected lists and their corresponding drug names, gene IDs, and UniProt ID results are listed in Table 1.

**Table 1:** Comparison results of connectivity score using LINCS L1000 dataset.

Cell line	Perturbagen ID	Drug name	Zhang score	Targets		
				Gene symbol	UniProt ID	Name of target proteins*
A375	BRD-K15933101	Ropinirole	0.152284551			
	BRD-K40758068	Efavirenz	0.14029383			
	BRD-K70505054	Ranitidine	0.127356285	*HRH2*	P25021	Histamine H2 receptor
A549	BRD-A64290322	Cyclosporine	0.151246937	*PPP3R2, PPIA*	Q96LZ3, P62937	Calcineurin subunit B type 2, Peptidyl-prolyl cis–trans isomerase A
	BRD-A79768653	Sirolimus	0.113739203	*MTOR*	P42345	Serine/threonine-protein kinase mTOR
	BRD-K84937637	Sirolimus	0.111655751	*MTOR*	P42345	Serine/threonine-protein kinase mTOR
MCF7	BRD-A22032524	Amlodipine	0.19968588	*CACNA1C, CACNA1I*	Q13936, Q9P0X4	Voltage-dependent L-type calcium channel subunit alpha-1C, Voltage-dependent T-type calcium channel subunit alpha-1I
	BRD-A01320529	Salmeterol	0.194945701			
	BRD-K91263825	Nortriptyline	0.182943722	*SLC6A2, SLC6A4, HTR2A*	P23975, P31645, P28223	Sodium-dependent noradrenaline transporter, Sodium-dependent serotonin transporter, 5-hydroxytryptamine receptor 2A
PC3	BRD-K89732114	Trifluoperazine	0.158636237	*DRD2, CALY, ADRA1A*	P14416, 9NYX4, P35348	Dopamine D2 receptor, Neuron-specific vesicular protein calcyon, Alpha-1A adrenergic receptor
	BRD-A45889380	Quinacrine	0.155439425	*PLA2G6, PLA2G4A, PLCL1*	O60733, P47712, Q15111	85/88 kDa calcium-independent phospholipase A2, Cytosolic phospholipase A2, Inactive phospholipase C-like protein 1
	BRD-A29485665	Bicalutamide	0.131271152	*AR*	P10275	Androgen receptor

*Filtered option: ‘antagonist or inhibitor, human protein, pharmacological action = yes’ were used.

**Figure 5:**
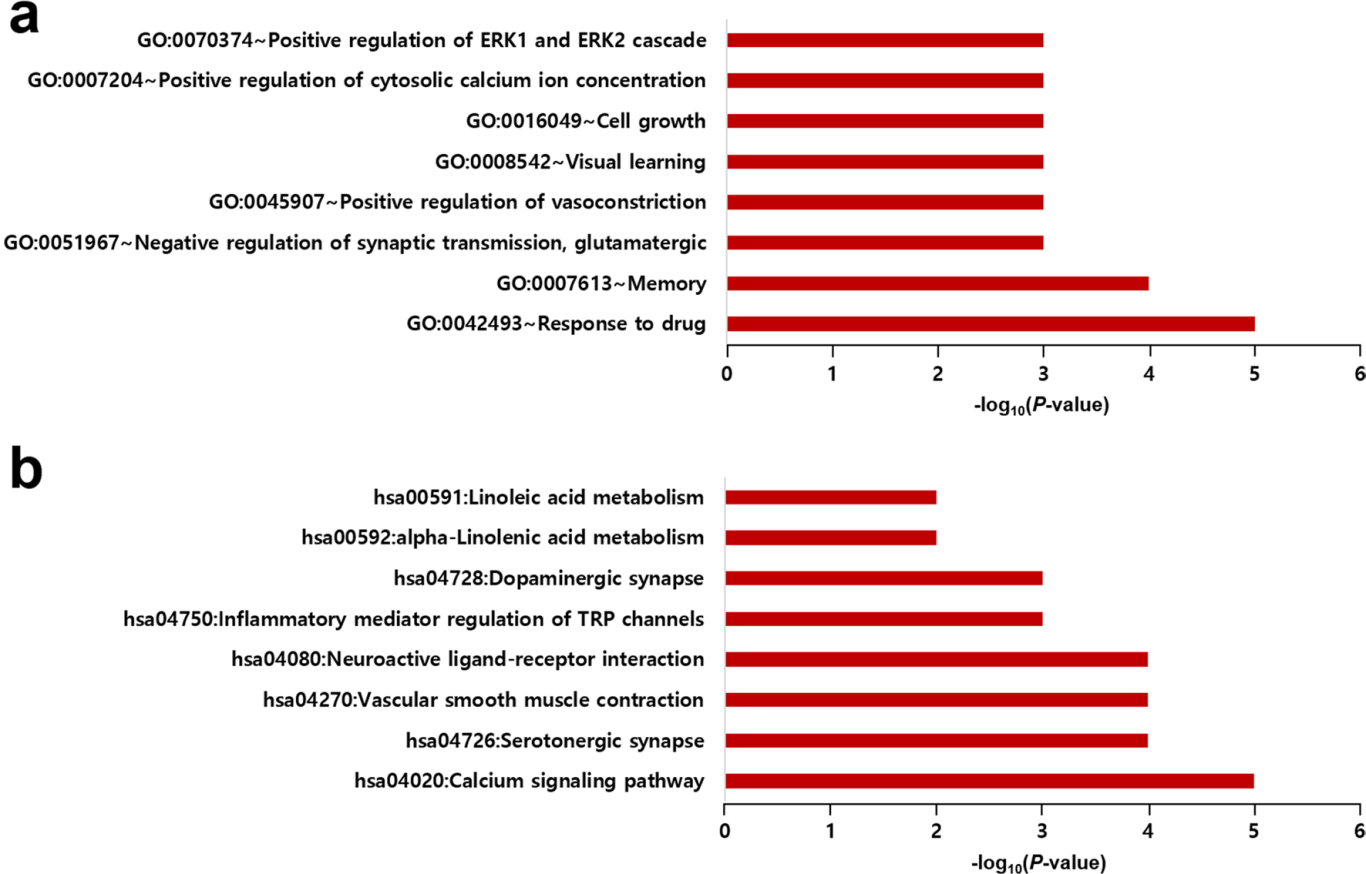
(**a**) GO and (**b**) KEGG pathway enrichment analysis of potential target genes.

In this study, we focused on drugs that could inhibit target proteins. The rationale behind this decision is that developing inhibitor drugs is a common approach, and developing an activator is more difficult than developing an inhibitor. Therefore, only human proteins that are inhibitor targets by approved drugs (i.e., antagonists and/or inhibitors) with known pharmacological action were considered in the DrugBank database^40^. A total of 16 valid proteins targeted by 12 approved drugs were obtained such as histamine H2 receptor (*HRH2*), calcineurin subunit B type 2 (*PPP3R2*), peptidyl-prolyl cis–trans isomerase A (*PPIA*), serine/threonine-protein kinase mTOR (*MTOR*), voltage-dependent L-type calcium channel subunit alpha-1C (*CACNA1C*), voltage-dependent T-type calcium channel subunit αalpha-1I (*CACNA1I*), sodium-dependent noradrenaline transporter (*SLC6A2*), sodium-dependent serotonin transporter (*SLC6A4*), 5-hydroxytryptamine receptor 2A (*HTR2A*), D(2) dopamine receptor (*DRD2*), neuron-specific vesicular protein calcyon (*CALY*), α-1A adrenergic receptor (*ADRA1A*), 85/88 kDa calcium-independent phospholipase A2 (*PLA2G6*), cytosolic phospholipase A2 (*PLA2G4A*), inactive phospholipase C-like protein 1 (*PLCL1*), and androgen receptor (*AR*). To identify the functions of 16 target proteins, GO enrichment and KEGG pathway enrichment analyses were performed, and a list with *p* < 0.05, as shown in Tables S6 and S7. BP GO terms were evenly distributed and affected the nervous system, such as the drug response to stimulation, memory, and synaptic transmission (Fig. 5a). In the KEGG pathway enrichment analysis, the distribution of calcium signaling pathways and serotonergic synapses was highly involved in neurotransmission pathway (Fig. 5b). From the results of GO enrichment and KEGG pathway enrichment analyses, it was shown that filtered candidate groups of target proteins generally inhibit the nervous system.

Among the 16 predicted targets, eight targets were identified to be capable of being used for the antagonist radioligand binding assays (*HRH2*, *CACNA1C*, *CACNA1I*, *SLC6A2*, *SLC6A4*, *HTR2A*, *DRD2*, and *ADRA1A*). *CACNA1I* was excluded because it is a gene belonging to the same family as *CACNA1C*. Therefore, we conducted an in vitro binding assay with seven predicted targets including H2 human histamine GPCR binding assay for the histamine H2 receptor (*HRH2*), Ca v1.2 human calcium ion channel binding assay for voltage-dependent L-type calcium channel subunit α-1C (*CACNA1C*), NET human norepinephrine transporter binding assay for sodium-dependent noradrenaline transporter (*SLC6A2*), SET human serotonin transporter binding assay for sodium-dependent serotonin transporter (*SLC6A4*), 5-HT2A human serotonin GPCR binding assay for 5-hydroxytryptamine receptor 2A (*HTR2A*), D2L human dopamine GPCR binding assay for D(2) dopamine receptor (*DRD2*), and α-1A human adrenoceptor GPCR binding assay for the α-1A adrenergic receptor (*ADRA1A*).

### Experimental validation of KR-37524 target proteins

**Figure 6:**
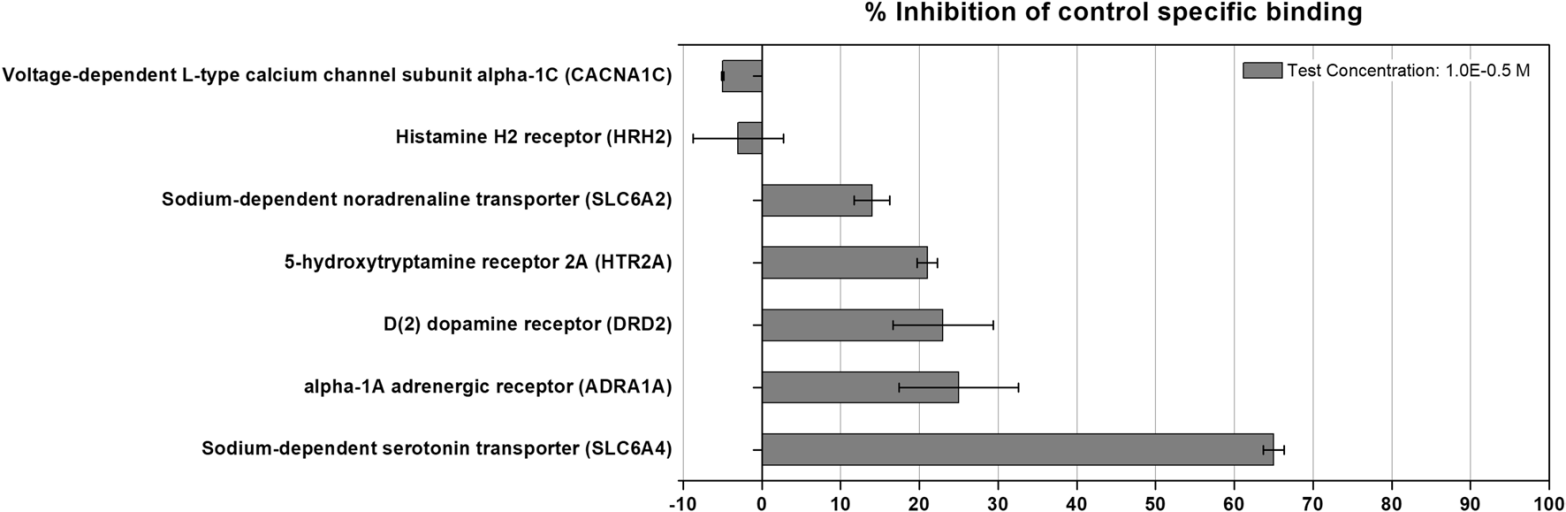
Experimental validation of potential targets of KR-37524.

KR-37524 was used to treat seven target proteins capable of antagonist radioligand testing to confirm that KR-37524 acts on the target protein we selected. As shown in Fig. 6, KR-37524 exhibited more than 50% interaction with the sodium-dependent serotonin transporter (*SLC6A4*). In contrast, less than 50% inhibition was observed for other targets at 10 µM KR-37524.

## Discussion

Urotensin-II receptor is a notable target for various cardiovascular diseases, such as heart failure, pulmonary hypertension, and atherosclerosis. However, owing to the unsatisfactory proof-of-concept and cardiotoxicity issues, numerous urotensin-II receptor antagonists have been discontinued during the development of new drugs for cardiovascular treatment. Unlike other discontinued drugs, we wanted to find new possibilities for KR-37524, which is known to be safe for toxicity issues. The drug repositioning approach involves expanding the indication of the targeted drug as well as altering the indication for a previously developed drug with a new function by changing the drug target. In general, finding a new target protein requires understanding not only the properties of the drug, but also the mechanism of the target proteins, although it can be difficult to identify a new target protein. Therefore, we applied a computational method to identify other effective target proteins to increase the usability of KR-37524.

Primarily, we identified the gene expression profile changes through microarray experiments after treatment with KR-37524 in four cancer cell lines to understand the cellular response to drug treatment. Next, we performed pathway enrichment analysis using the DEGs after treatment with KR-37524. KR-37524 induced the gene expressions involved in cholesterol synthesis and metabolism, and KR-37524 reduced the gene expressions involved in cell differentiation and the nervous system. The regulation of cholesterol synthesis is known to be regulated by UTR^41–43^. Although pathway enrichment analysis using transcriptome data reveals the regulatory mechanisms of drugs at the pathway level, it has limitations in identifying the direct inhibitory targets of the compound.

Therefore, we used another approach to compare the experimental information and microarray data from KR-37524 with that in the LINCS L1000 database. The LINCS L1000 data measures the gene expression levels of 978 landmark genes, and the gene expression levels of other genes were estimated using a computational model. LINCS L1000 includes gene expression profile changes induced by thousands of perturbagens in various cell lines. Therefore, it was convenient to compare the gene expression profile changes induced by KR-37524. This enables comparative analyses of several experimental groups at the same time to identify any differences between them. Identification of approved drugs with similar gene expression profile changes in LINCS L1000 to that induced by KR-37524 could indirectly determine a shared target protein targeted by KR-37524. Since the LINCS L1000 dataset contains most of the genes expressed in cells, this served our goal of finding other target proteins; landmark genes were selected as genes that are widely expressed in cells, while the expression of other genes that were not directly measured in the analysis could be inferred^19^. Thus, by comparing the LINCS L1000 dataset with the KR-37524 data, it was possible to find drugs for a new target.

The connectivity score was used to identify the most similar gene expression profile changes between the two datasets. In this study, we used the Zhang score, an improved version of the connectivity score, CMap^39^. Since we have already used the landmark gene to find other targets in the cell, we applied the Zhang score, which assigns more weight to the most differentially expressed genes. This method is appropriate for searching for drugs with similar gene expression profile changes, and drug candidates were selected by sorting by a high score. In the DrugBank database, we searched for a list of drug-target proteins and narrowed down those acting as inhibitors. As a result of performing GO enrichment and KEGG pathway enrichment analysis using the gene list of target proteins we selected, the drug information for the nervous system, such as response to drug and negative regulation of synaptic transmission, was retrieved. In the KEGG pathway enrichment analysis, pathways such as calcium signaling and the serotonergic synapse pathway were selected. Thus, we predicted that KR-37524 could act on serotonin-related target proteins. Finally, receptor-binding experiments confirmed that KR-37524 has a high affinity for human serotonin transporters.

Our approach compared gene expression profile changes with the approved drugs in the LINCS L1000 database. In this case, it is possible to screen only well-known target proteins of approved drugs. Therefore, structural modeling technique based on machine learning has been recently proposed as a way to overcome this problem. Machine learning-based technique is accurate enough to predict most human protein structures^44^. This is expected to provide a great opportunity for drug repositioning by predicting more diverse target proteins.

## Conclusion

We used a transcriptome-based drug repositioning approach to identify new target proteins for KR-37524. Although other effects of KR-37524 were unknown, we were able to effectively infer the characteristics of KR-37524 represented as gene expression profile changes affecting potential target proteins from the analysis of transcriptome data. The LINCS L1000 database provides drug-induced transcriptome data (i.e., gene expression profile changes induced by perturbagens) under a variety of conditions, which we utilized to help identify new target proteins. In particular, by taking only filtered data from specific experimental conditions, gene expression profile changes could be appropriately applied. The in vitro binding assay for KR-37524 against seven candidate target proteins selected by connectivity score was performed to confirm its potential as a new target. Consequently, the serotonin transporter was identified as a novel target. This is expected to provide a new function to KR-37524 in addition to the indicated treatment for cardiovascular disease. The standalone software, which was used for drug repositioning, effectively suggested potential target proteins, and the LINCS L1000 database is easily accessible to researchers. Therefore, our approach is expected to be valuable for drug repositioning research.

## Materials and methods

### Materials

KR-37524 (CAS Registry No. 2228942-83-2), (4-(3-bromo-4-(piperidin-4-yloxy)benzyl)-*N*-(3-(dimethylamino)phenyl) piperazine-1-carboxamide dihydrochloride), was synthesized at the Research Center for Medicinal Chemistry, Korea Research Institute of Chemical Technology (KRICT, Daejeon, Korea). Four cell lines, human skin malignant melanoma cells (A375), human lung carcinoma cells (A549), human breast carcinoma cells (MCF7), and human prostate adenocarcinoma cells (PC3) were purchased from American Type Culture Collection (ATCC, Rockville, MD, USA).

### Cell culture and sample preparation

A375 and A549 cells were cultured in RPMI-1640 supplemented with 10% fetal bovine serum (FBS) and 1% penicillin–streptomycin–glutamine. MCF7 cells were cultured in Dulbecco's modified Eagle's medium (DMEM) with 100% FBS and 1% penicillin–streptomycin–glutamine. PC3 cells were cultured in RPMI with 10% FBS, 1% penicillin–streptomycin–glutamine, 1 mM sodium pyruvate, and 10 mM 4-(2-hydroxyethyl)-1-piperazineethanesulfonic acid (HEPES).

The cells were first incubated for two weeks to stabilize after initial seeding. KR-37524 dissolved in DMSO was stored at 10 nM at − 80℃. Cell lines were seeded in six 60-mm dishes (DMSO × 3 and KR-37524 × 3). Cells were plated at 8.45 × 10^5^ cells for MCF7, 1.496 × 10^6^ cells for PC3, 1.1375 × 10^6^ cells for A375, and 1.3 × 10^6^ cells for A549 under the culture conditions. After 24 h, DMSO and KR-37524 were diluted 1000-fold and used to treat the cells. The final concentrations of DMSO and KR-37524 were 0.1% and 10 μM, respectively. After 6 h, total RNA from these cells was isolated using 1 mL of TRIzol reagent. After cell destruction by pipetting, the cells were stored in a deep freezer (-80℃).

### Microarray data analysis

Total RNA samples were assessed using the Clariom™ S Assay, Human platform. cDNA was synthesized using the GeneChip WT (Whole Transcript) Amplification kit, as described by the manufacturer. Sense cDNA was then fragmented and biotin-labeled with TdT (terminal deoxynucleoridyl transferase) using the GeneChip WT Terminal labeling kit. Approximately 5.5 μg of labeled DNA target was hybridized to the Affymetrix GeneChip Array at 45 ℃ for 16 h. Hybridized arrays were washed and stained on a GeneChip Fluidics Station 450 and scanned using a GCS3000 scanner.

Microarray data export processing and basic analysis were performed using the Affymetrix^®^ GeneChip Command Console® Software version 6.0 + (AGCC, www.thermofisher.com/kr/ko/home/life-science/microarray-analysis/microarray-analysis-instruments-software-services/microarray-analysis-software/affymetrix-genechip-command-console-software.html). The data were summarized and normalized using the signal space transformation-robust multichip analysis (SST-RMA) method implemented in Affymetrix^®^ Power Tools version 2.11.4 (APT, www.thermofisher.com/kr/en/home/life-science/microarray-analysis/microarray-analysis-partners-programs/affymetrix-developers-network/affymetrix-power-tools.html). We exported the results with gene level SST-RMA analysis. The statistical significance of the expression data was determined using an independent t-test and fold change, in which the null hypothesis was that no difference exists among groups. The fold change and *p* value cut-off are 1.5 and 0.05, respectively.

The Database for Annotation, Visualization, and Integrated Discovery (DAVID) functional annotation tools^37,38^ were used to calculate gene enrichment, pathways, and functional annotation analysis for a significant probe list.

### LINCS L1000 database search and candidate target protein selection

The LINCS L1000 database was used to compare the gene expression profile changes induced by KR-37524 treatment with the gene expression profile changes induced by thousands of perturbagens obtained from various times, points, doses, and cell lines. The LINCS L1000 level 5 dataset, perturbation, signature, and landmark gene lists were downloaded from the Gene Expression Omnibus (GEO; GSE92742) (https://www.ncbi.nlm.nih.gov/geo/query/acc.cgi?acc=GSE92742). The LINCS L1000 level 5 dataset contains gene expression profile changes of 978 landmark genes, which were measured directly by the L1000 assay. LINCS L1000 raw data were filtered by the cell line type and gene expression level under the same conditions as those for KR-37524 treatment for 6 h at a dose of 10 μM. Fold change was calculated by obtaining the mean value of the sample gene expression level per cell line of KR-37524, and dividing this by the mean value of DMSO. The fold change was then normalized using log_2_ and compared with the LINCS fold change of 978 landmark gene expression patterns. We used the DrugBank drug list to find a list of approved drugs in the LINCS database. We collected the simplified molecular input line entry system (SMILES) of drugs from the LINCS and DrugBank, and calculated the Tanimoto coefficient^45^ to list the drugs that matched perfectly (Tanimoto coefficient = 1.0).

The connectivity score (Zhang score) was calculated to find LINCS L1000 data similar to the gene expression profile changes induced by KR-37524. To compare gene expression patterns, Lamb et al. created a CMap^16^, converted the gene expression patterns of known chemicals into a database, and then calculated an order according to the expression level of test DEGs. Based on this, Zhang et al. developed a simple and more accurate and sensitive method to calculate the connection between two gene expression profile changes^39^. The connectivity score has a value between − 1 and 1, where 1 indicates the maximum positive connection strength with the reference profile, whereas − 1 indicates that two experimental perturbations had the maximum inverse correlation. All program scripts used in this calculation are available in the web repository (https://bitbucket.org/krictai/lincs_search).

### In vitro binding assay for target validation

The receptor binding affinity of KR-37524 was determined by profiling services at Eurofins Cerep (Test No.: FR095-0019211, US034-0011488; I’Evescault, France) using radioligand binding assays for seven distinct human receptors and transporters; α-1A adrenergic receptor (*ADRA1A*), D(2) dopamine receptor (*DRD2*), histamine H2 receptor (*HRH2*), sodium-dependent noradrenaline transporter (*SLC6A2*), 5-hydroxytryptamine receptor 2A (*HTR2A*), sodium-dependent serotonin transporter (*SLC6A4*), and voltage-dependent L-type calcium channel subunit α-1C (*CACNA1C*). To evaluate the percentage (%) inhibition of specific binding, all radioligand binding assays were performed in 96 well plates at 37℃ in binding buffer (25 mM HEPES, 100 mM NaCl, 2 mM MgCl_2_, and 1 mM 3-[(3-cholamidopropy) dimethyl ammonio]-1-propanesulfonate [CHAPS] at pH 7.4 [NaOH]). The human recombinant receptor membranes of the α-1A adrenergic receptor (*ADRA1A*), sodium-dependent noradrenaline transporter (*SLC6A2*), 5-hydroxytryptamine receptor 2A (*HTR2A*), and sodium-dependent serotonin transporter (*SLC6A4*) were used in the CHO cell membrane, and D(2) dopamine receptor (*DRD2*), histamine H2 receptor (*HRH2*), and voltage-dependent L-type calcium channel subunit α-1C (*CACNA1C*) were used in the HEK293 cell membrane overexpressed by each receptor. The specific radiolabeled ligands of α-1A adrenergic receptor (*ADRA1A*), D(2) dopamine receptor (*DRD2*), histamine H2 receptor (*HRH2*), sodium-dependent noradrenaline transporter (*SLC6A2*), 5-hydroxytryptamine receptor 2A (*HTR2A*), sodium-dependent serotonin transporter (*SLC6A4*), and voltage-dependent L-type calcium channel subunit α-1C (*CACNA1C*) were [^3^H]prazosin, [^3^H]methyl-spiperone, [^125^I]APT, [^3^H]nisoxetine, [^3^H]ketanserin, [^3^H]imipramine, and [^3^H]PN200-110, respectively. Nonspecific binding was defined in the presence of 0.1 mM epinephrine, 10 μM butaclamol, 100 μM tiotidine, 1 μM desipramine, 1 μM ketanserin, 10 μM imipramine, and 1 μM isradipine, respectively. Ligand binding was determined by filtration of the assay mixture over GF/C Whatman filters (Cytiva, Marlborough, MA, USA). After washing the filters, liquid scintillation counting was performed to quantify the radioactivity. Compound binding was calculated as the % inhibition of the binding of a radioactively labeled ligand specific to each target. In each experiment, and when applicable, the respective reference compound was tested concurrently with KR-37524, and the data were compared with historical values determined at Eurofins. The experiment was performed in accordance with the Eurofins validation standard operating procedure. The receptor binding assays were performed in triplicate, and each result was determined in three independent experiments.

## Supplementary Information

Supplementary Information.

## Data Availability

Standalone software (https://bitbucket.org/krictai/lincs_search) used in the current study to generalize our strategy. Microarray data of four cell lines (A375, A549, MCF7, and PC3) treated with KR-37524 are available in Gene Expression Omnibus (GEO), accession number is GSE181088 (https://www.ncbi.nlm.nih.gov/geo/query/acc.cgi?acc=GSE181088).
