# Supplementary material for: Identification of new target proteins of a Urotensin-II receptor antagonist using transcriptome-based drug repositioning approach
Source: Sci Rep. 2021 Aug 24;11:17138. doi: 10.1038/s41598-021-96612-0 (PMC8384862; doi:10.1038/s41598-021-96612-0)
Supplement: Supplementary file 1 — Supplementary Information. [file 41598_2021_96612_MOESM1_ESM.docx]

Table S1. The shared and unique genes of Venn diagram.

|  | **Position (Counts)** | **Genes** |
| --- | --- | --- |
| **Upregulated genes** | A375 (75) | *ACTL7B, ALS2, APOBEC3A, ARHGEF9, BCAT1, BLOC1S5, C20orf196, C7orf57, C9orf152, CCL13, CEP120, CLDN12, COL9A1, CTBP2, CYP1A2, DET1, DYNLT3, EPT1, FAM72A, FAM72B, FAM72C, FANCL, FBXO45, GK2, GPR180, GRIA4, HOXD12, KIAA1107, KIDINS220, KLHL9, LEFTY1, LNP1, MTM1, NEK7, NEUROG2, NUP107, OR51G1, PGM2L1, PKHD1, PNPT1, PPP1R12B, PTPRT, RANBP2, RBM7, RFPL4B, RNF185, SAMD5, SCN8A, SENP5, SESN3, SLC25A21, SNAI3, SPRY3, SYCP2L, TBC1D8B, TM2D1, TMEM167A, TMEM263, TRHDE-AS1, TRPS1, TSPAN5, UBA6, UTS2R, WFDC10B, ZAR1, ZBTB6, ZDHHC17, ZNF132, ZNF195, ZNF208, ZNF430, ZNF441, ZNF567, ZNF701, ZNF808* |
|  | A549 (57) | *ACSL4, ANKRD35, AOC2, ARHGAP1, ASXL3, CACNA1E, CCDC85A, CCNI2, CLCN6, CNTD1, DHCR24, ELMOD1, EMX2, FASN, FDFT1, FFAR2, FNIP1, GIGYF1, GNPDA1, GP1BB, HOXC13, HSD17B7, IDH1, KCTD11, KIRREL3, LEKR1, LPXN, LSS, MARCH10, MFSD14A, MVK, MZF1, NEK5, OR4A5, PDE4DIP, PNLDC1, PTCHD3, RASA4, SLC25A30, SLC29A2, SOX21, SREBF2, TBC1D17, TBC1D22A-AS1, THAP8, TRDN, TSPAN2, TTC25, UBE2U, ULBP1, USH2A, WNT5B, ZNF395, ZNF513, ZNF618, ZNF678, ZNF821* |
|  | MCF7 (96) | *ABCG4, ACSS3, ATP10A, AWAT1, BCL2L14, BCO1, BEST2, BLM, C10orf126, C3orf49, C5orf28, CACFD1, CACNA2D4, CAMK1G, CBLN3, CCDC7, CHD2, CIB3, CNPY4, CRNDE, CTGF, DCP2, DDO, DDR2, DNAH10, DYRK4, EIF2AK3, EPG5, EXOC4, FAM25G, FAM45A, FBXO32, FKBP7, GBP4, GHRHR, GLRB, GLRX, GPR75, GPX8, INPP5D, ITGB1BP2, LDHAL6B, LINC01590, LRFN2, LRRC29, MON1A, MRVI1, NDUFA10, NEU2, NME9, OR10W1, OR1D4, OR1G1, OR51I2, OR6C74, OXCT1, P2RX6, PATE1, PLCL2, PLEKHA6, POTEC, PRICKLE1, PRKG2, PRND, PSAT1, RAB39B, RAB43, REXO2, RFX4, RFX8, RTP3, SETBP1, SLC23A3, SLC24A2, SLC38A9, SLC4A5, SMYD3, SRP72, ST8SIA1, TAGLN, TBX6, TCERG1L, TCP10L, TEX29, TRIP12, TRPV2, TTC6, USP34, USP47, VIM-AS1, VLDLR, YBX1, ZBTB34, ZNF540, ZNF713, ZPBP2* |
|  | PC3 (135) | *ADAM23, AKAP11, ALG10, ALG13, APAF1, APIP, ATF7IP2, BCHE, BEAN1, BRIP1, BRWD1, C1orf131, CBR4, CCDC109B, CCDC141, CD9, CDH13, CEP135, CLTCL1, COLEC10, CREBZF, CRLF3, CST11, CTAG1B, CTAGE6, CTAGE8, CYBRD1, DNAJB11, DNAJB9, DNAJC3, DOK2, DTWD2, DUSP15, EDEM3, EFCAB13, FABP2, FABP4, FAM135A, FAM174A, FAM3C2, FEM1C, FPGT, FPGT-TNNI3K, FSD1L, GABPA, GAL3ST4, GFPT1, GPATCH11, GRAMD3, HEBP1, HERPUD1, HIBCH, HSP90B1, HSPA5, IL18R1, ITGA11, JAK2, KCNMB3, KDM3A, KIAA1109, KLHL23, KLRAP1, KLRG1, KRAS, KRIT1, LCA5, LRP6, LSM6, LY6E, LYRM7, MAATS1, MAN2A2, MEGF11, MEGF9, MEIS2, MICALCL, MLLT3, MRFAP1L1, MTMR3, NBEAL1, NIPAL1, NPR3, NRP1, NUDT21, OR2T3, PDIA4, PI4K2B, PIGA, PIK3CB, PJA2, PKD2, PLEKHA5, POLI, PSG1, PSG7, PUS7L, RAB33B, RASGRF2, RLIM, RNASE2, RNMT, RWDD3, SDF2L1, SF3B6, SGK3, SLC33A1, SPAM1, SYNM, TBC1D32, TBC1D3P5, TBCA, TCEA1, TMCC3, TMEM135, TMEM50B, TNFSF13B, TRMT12, TRNT1, TROVE2, TRPM7, TXNDC11, UGCG, USP53, VPS13A, WDR89, ZBTB41, ZBTB5, ZKSCAN5, ZNF197, ZNF215, ZNF33B, ZNF385D, ZNF608, ZNF654, ZNF682* |
|  | A375 ∩ A549 (6) | *APOL1, CLIC4, DHCR7, ELOVL6, MSMO1, ZNF718* |
|  | A375 ∩ MCF7 (0) |  |
|  | A375 ∩ PC3 (10) | *ELF1, ESCO1, EXOC6, MOSPD2, OSTM1, PDGFRL, PPIP5K2, RAD21, STXBP5, TMEM41B* |
|  | A549 ∩ MCF7 (0) |  |
|  | A549 ∩ PC3 (2) | *IDI1, SCD* |
|  | MCF7 ∩ PC3 (9) | *ABCA5, CCDC186, DUSP16, EID3, GPR151, KCTD7, RAPGEF6, TXNDC15, VAPA* |
|  | A375 ∩ A549 ∩ MCF7 (1) | *HMGCS1* |
|  | A375 ∩ MCF7 ∩ PC3 (1) | *SLC7A11* |
|  | A375 ∩ A549 ∩ PC3 (3) | *INSIG1, LDLR, STARD4* |
|  | A549 ∩ MCF7 ∩ PC3 (0) |  |
|  | A375 ∩ A549 ∩ MCF7 ∩ PC3 (3) | *HMGCR, LPIN1, SQLE* |
| **Downregulated genes** | A375 (97) | *ABHD11, ABHD17A, AC010894.3, ACOT13, ANAPC11, APBB1, APLF, APOC1, APRT, ARL13A, ATP5I, AURKAIP1, BPHL, C11orf1, C11orf84, C12orf10, C1orf53, CDC34, CDK5R1, CEACAM18, CFAP54, CLTB, COA6, COMMD9, CXCL3, DEFB107B, DRAP1, EMP2, FAM150B, FAM222A, FIS1, FUZ, GAPT, GPX1, HIGD2A, HIST2H2AB, IGFBP7, INHBC, KRTAP2-3, LGALS1, LINC00493, LSM2, LSM3, MAL, MFSD10, MFSD3, MGST3, MPG, MRPL4, MTHFS, MZT2B, NAA11, NAA38, NAPRT, NDUFA1, NDUFA13, NDUFS7, NPNT, OR14I1, OR56A1, OR5H15, PAIP2B, PET100, PEX16, PFDN6, PKIG, POLR2F, POLR2I, PSCA, PSTK, RNF26, ROPN1L, RRP7A, S100A16, SEPW1, SHISA4, SIDT2, SMDT1, SMUG1, SRA1, ST6GAL2, ST6GALNAC6, STIM2, TFF1, TIMM8B, TNFAIP6, TOE1, TST, UBAP1L, UBE2S, UQCR11, VMAC, VPS28, WNT11, XRCC3, YIF1B, ZNF503* |
|  | A549 (42) | *AZU1, C1orf185, CIITA, COL10A1, CSF1R, DDX5, DEFB106B, DENND3, DNAI1, DSG3, DUSP16, EPB42, FAM153A, FGFR1OP2, FRMPD3, GCNT7, GJA4, GNG8, GOLGA6L10, GPC6, GPR183, GRIN2A, GRK5, HSD3B1, IGSF9B, IL20RA, KBTBD3, LOC100130691, LOXL1, MLXIP, MRAS, NEUROD2, PTPRR, SERPINC1, SPINK14, SRPX, TCF7, TRAPPC4, TRIM27, UBA7, USP34, ZMYM2* |
|  | MCF7 (61) | *ADAM28, ARHGAP1, BIRC3, BIRC7, C1orf198, CA5A, CARHSP1, CISH, CLCF1, CST3, CYB561, CYSLTR1, DGCR2, DOLPP1, DUSP5, ELK1, FAM132B, FANCG, FBRSL1, FOXO6, GNAO1, GPN2, GTSF1, HOXC13, HS6ST3, HYOU1, JUNB, LFNG, LOC101928137, LSR, MATK, MED25, MYC, NDRG1, OR2AP1, PCIF1, PCYOX1L, PPIF, PSRC1, RAET1L, RFFL, SERPINA5, SEZ6L, SLC25A29, SLC8B1, SLMAP, SPATA31D1, SPRR3, TBC1D10A, TMEM45B, TRIM49B, TSPAN9, TTC22, URGCP, VPS9D1, XPNPEP3, ZBTB7B, ZNF304, ZNF362, ZNF703, ZYX* |
|  | PC3 (76) | *ABCC3, ADAMTS7, ADCK4, ADH1A, ARHGEF25, ARRDC1, ART3, ASB12, C10orf55, C19orf70, C9orf84, CABP5, CATSPER1, CCBL1, CCDC85B, CDHR3, CHRNB3, CLCN2, COL3A1, COMMD4, CORO2B, CRHR1, E2F4, EIF4A3, EXOC3-AS1, FAM195A, GLYCTK, GP2, GTF2H2, HHLA1, HINT1, HMBS, IL11RA, ISG15, JMJD4, KCNN4, KNDC1, KRTAP10-9, LINC01537, MAP6D1, MED21, MRPS12, MTRNR2L10, NAP1L2, NDUFV3, NECAB2, NPTX1, OR4C11, ORAI1, PAQR7, PDE3B, PIGU, PLEKHG4B, PPARA, PRB4, PRRT3, R3HCC1, R3HDM4, RAB43, RAB4B, RPP25L, RTKN, SECTM1, SERPINA12, SERTAD1, SIL1, SMAD7, SRGN, TAF6, TM4SF19, TMEM38A, TNS2, UBAC1, VASH2, VSIG4, VWA5A* |
|  | A375 ∩ A549 (2) | *COMMD6, FKBP2* |
|  | A375 ∩ MCF7 (0) |  |
|  | A375 ∩ PC3 (4) | *C8orf59, ID3, MFSD5, S100A13* |
|  | A549 ∩ MCF7 (0) |  |
|  | A549 ∩ PC3 (0) |  |
|  | MCF7 ∩ PC3 (1) | *DPYSL4* |
|  | A375 ∩ A549 ∩ MCF7 (0) |  |
|  | A375 ∩ MCF7 ∩ PC3 (0) |  |
|  | A375 ∩ A549 ∩ PC3 (0) |  |
|  | A549 ∩ MCF7 ∩ PC3 (0) |  |
|  | A375 ∩ A549 ∩ MCF7 ∩ PC3 (0) |  |

Table S2. Gene list for each cell line of GO enrichment analysis

|  | **Cell line** | **Biological Process (BP)** | **Counts** | **%** | ***p*-value** | **Genes** |
| --- | --- | --- | --- | --- | --- | --- |
| **Upregulated genes** | A375 | GO:0006695~cholesterol biosynthetic process | 6 | 6.67 | 6.94637E-07 | *DHCR7, MSMO1, INSIG1, SQLE, HMGCR, HMGCS1* |
|  |  | GO:0008203~cholesterol metabolic process | 4 | 4.44 | 0.003482739 | *APOL1, LDLR, INSIG1, SQLE* |
|  |  | GO:0007077~mitotic nuclear envelope disassembly | 3 | 3.33 | 0.016497127 | *LPIN1, NUP107, RANBP2* |
|  |  | GO:0070508~cholesterol import | 2 | 2.22 | 0.026504921 | *LDLR, STARD4* |
|  |  | GO:0016126~sterol biosynthetic process | 2 | 2.22 | 0.043788538 | *MSMO1, SQLE* |
|  | A549 | GO:0006695~cholesterol biosynthetic process | 12 | 17.65 | 6.20998E-19 | *DHCR7, MSMO1, HSD17B7, DHCR24, INSIG1, FDFT1, LSS, SQLE, HMGCR, MVK, HMGCS1, IDI1* |
|  |  | GO:0055114~oxidation-reduction process | 10 | 14.71 | 0.000267147 | *DHCR7, MSMO1, HSD17B7, DHCR24, SCD, FDFT1, FASN, SQLE, AOC2, HMGCR* |
|  |  | GO:0008299~isoprenoid biosynthetic process | 5 | 7.35 | 1.53508E-07 | *FDFT1, HMGCR, MVK, HMGCS1, IDI1* |
|  |  | GO:0008203~cholesterol metabolic process | 5 | 7.35 | 0.000107868 | *APOL1, SREBF2, LDLR, INSIG1, SQLE* |
|  |  | GO:0035338~long-chain fatty-acyl-CoA biosynthetic process | 4 | 5.88 | 0.00047338 | *SCD, FASN, ELOVL6, ACSL4* |
|  |  | GO:0006629~lipid metabolic process | 4 | 5.88 | 0.019433761 | *SREBF2, LDLR, HMGCS1, ACSL4* |
|  |  | GO:1902476~chloride transmembrane transport | 3 | 4.41 | 0.044969058 | *APOL1, CLCN6, CLIC4* |
|  |  | GO:0033489~cholesterol biosynthetic process via desmosterol | 2 | 2.94 | 0.014453026 | *DHCR7, DHCR24* |
|  |  | GO:0033490~cholesterol biosynthetic process via lathosterol | 2 | 2.94 | 0.014453026 | *DHCR7, DHCR24* |
|  |  | GO:0042759~long-chain fatty acid biosynthetic process | 2 | 2.94 | 0.021602291 | *SCD, ELOVL6* |
|  |  | GO:0070508~cholesterol import | 2 | 2.94 | 0.021602291 | *LDLR, STARD4* |
|  |  | GO:2000188~regulation of cholesterol homeostasis | 2 | 2.94 | 0.032230642 | *SREBF2, LDLR* |
|  |  | GO:0016126~sterol biosynthetic process | 2 | 2.94 | 0.035748126 | *MSMO1, SQLE* |
|  |  | GO:0008610~lipid biosynthetic process | 2 | 2.94 | 0.049692744 | *FDFT1, ACSL4* |
|  | MCF7 | GO:0070588~calcium ion transmembrane transport | 4 | 4.12 | 0.01735927 | *SLC24A2, CACNA2D4, CACFD1, TRPV2* |
|  |  | GO:0051260~protein homooligomerization | 4 | 4.12 | 0.04778778 | *PRND, P2RX6, KCTD7, EIF2AK3* |
|  |  | GO:0006695~cholesterol biosynthetic process | 3 | 3.09 | 0.013113642 | *SQLE, HMGCR, HMGCS1* |
|  |  | GO:0045454~cell redox homeostasis | 3 | 3.09 | 0.0486929 | *TXNDC15, NME9, GLRX* |
|  |  | GO:0035988~chondrocyte proliferation | 2 | 2.06 | 0.040530204 | *CTGF, DDR2* |
|  | PC3 | GO:0036498~IRE1-mediated unfolded protein response | 5 | 3.29 | 0.001029762 | *DNAJB9, GFPT1, DNAJB11, DNAJC3, HSPA5* |
|  |  | GO:0006695~cholesterol biosynthetic process | 4 | 2.63 | 0.002937866 | *INSIG1, SQLE, HMGCR, IDI1* |
|  |  | GO:0045454~cell redox homeostasis | 4 | 2.63 | 0.02059458 | *TXNDC11, TXNDC15, PDIA4, KRIT1* |
|  |  | GO:0008033~tRNA processing | 3 | 1.97 | 0.031812301 | *PUS7L, LSM6, TRMT12* |
|  |  | GO:0051262~protein tetramerization | 3 | 1.97 | 0.036717343 | *NUDT21, HMGCR, TRPM7* |
|  |  | GO:0008542~visual learning | 3 | 1.97 | 0.045484605 | *HMGCR, MEIS2, KRAS* |
|  |  | GO:0070508~cholesterol import | 2 | 1.32 | 0.044535848 | *LDLR, STARD4* |
| **Downregulated genes** | A375 | GO:0006120~mitochondrial electron transport, NADH to ubiquinone | 3 | 2.97 | 0.025534451 | *NDUFS7, NDUFA13, NDUFA1* |
|  |  | GO:0032981~mitochondrial respiratory chain complex I assembly | 3 | 2.97 | 0.040528188 | *NDUFS7, NDUFA13, NDUFA1* |
|  |  | GO:0098869~cellular oxidant detoxification | 3 | 2.97 | 0.048998296 | *SEPW1, GPX1, MGST3* |
|  |  | GO:0043534~blood vessel endothelial cell migration | 2 | 1.98 | 0.049494691 | *EMP2, GPX1* |
|  | A549 | GO:0018108~peptidyl-tyrosine phosphorylation | 3 | 8.33 | 0.030368891 | *ZMYM2, CSF1R, FGFR1OP2* |
|  |  | GO:0007399~nervous system development | 3 | 8.33 | 0.092645777 | *NEUROD2, IGSF9B, GNG8* |
|  |  | GO:0045348~positive regulation of MHC class II biosynthetic process | 2 | 5.56 | 0.014206411 | *CIITA, AZU1* |
|  |  | GO:0045124~regulation of bone resorption | 2 | 5.56 | 0.015968435 | *CSF1R, IL20RA* |
|  |  | GO:0071257~cellular response to electrical stimulus | 2 | 5.56 | 0.021236258 | *NEUROD2, CIITA* |
|  |  | GO:0030316~osteoclast differentiation | 2 | 5.56 | 0.040319378 | *CSF1R, GPR183* |
|  | MCF7 | GO:0030154~cell differentiation | 5 | 8.62 | 0.05128266 | *SPATA31D1, ELK1, ZBTB7B, MATK, GTSF1* |
|  |  | GO:0008284~positive regulation of cell proliferation | 5 | 8.62 | 0.052626813 | *ZNF703, MATK, CST3, CLCF1, MYC* |
|  |  | GO:0006366~transcription from RNA polymerase II promoter | 5 | 8.62 | 0.069860455 | *HOXC13, ELK1, ZBTB7B, MYC, JUNB* |
|  |  | GO:0042493~response to drug | 4 | 6.90 | 0.064792488 | *CST3, GNAO1, MYC, JUNB* |
|  |  | GO:0001541~ovarian follicle development | 3 | 5.17 | 0.007205522 | *LFNG, FANCG, MYC* |
|  |  | GO:0009314~response to radiation | 2 | 3.45 | 0.081706801 | *FANCG, JUNB* |
|  |  | GO:0048147~negative regulation of fibroblast proliferation | 2 | 3.45 | 0.090062813 | *MYC, MED25* |
|  |  | GO:0002931~response to ischemia | 2 | 3.45 | 0.095592027 | *HYOU1, PPIF* |
|  | PC3 | GO:0006884~cell volume homeostasis | 2 | 2.70 | 0.031126335 | *KCNN4, E2F4* |

Table S3. Gene list for each cell line of KEGG pathway enrichment analysis

|  | **Cell line** | **Pathway** | **Counts** | **%** | ***p*-value** | **Genes** |
| --- | --- | --- | --- | --- | --- | --- |
| **Upregulated genes** | A375 | hsa01100:Metabolic pathways | 11 | 12.2 | 0.028986522 | *DHCR7, MSMO1, MTM1, SQLE, GK2, HMGCR, HMGCS1, EPT1, BCAT1, CYP1A2, LPIN1* |
|  |  | hsa01130:Biosynthesis of antibiotics | 5 | 5.56 | 0.012817389 | *MSMO1, SQLE, HMGCR, HMGCS1, BCAT1* |
|  |  | hsa04120:Ubiquitin mediated proteolysis | 4 | 4.44 | 0.021189771 | *DET1, UBA6, FANCL, KLHL9* |
|  |  | hsa00100:Steroid biosynthesis | 3 | 3.33 | 0.003327206 | *DHCR7, MSMO1, SQLE* |
|  | A549 | hsa01100:Metabolic pathways | 17 | 25.00 | 1.57396E-06 | *LSS, SQLE, AOC2, HMGCS1, MVK, LPIN1, DHCR7, HSD17B7, MSMO1, DHCR24, FDFT1, FASN, IDH1, HMGCR, GNPDA1, IDI1, ACSL4* |
|  |  | hsa01130:Biosynthesis of antibiotics | 10 | 14.71 | 6.12718E-08 | *MSMO1, HSD17B7, FDFT1, LSS, SQLE, HMGCR, IDH1, MVK, HMGCS1, IDI1* |
|  |  | hsa00100:Steroid biosynthesis | 7 | 10.29 | 7.53123E-11 | *DHCR7, MSMO1, HSD17B7, DHCR24, FDFT1, LSS, SQLE* |
|  |  | hsa00900:Terpenoid backbone biosynthesis | 4 | 5.88 | 7.90303E-05 | *HMGCR, MVK, HMGCS1, IDI1* |
|  |  | hsa01212:Fatty acid metabolism | 4 | 5.88 | 0.000829236 | *SCD, FASN, ELOVL6, ACSL4* |
|  |  | hsa04146:Peroxisome | 3 | 4.41 | 0.041547443 | *IDH1, MVK, ACSL4* |
|  | MCF7 | hsa01130:Biosynthesis of antibiotics | 5 | 5.15 | 0.028545418 | *LDHAL6B, SQLE, PSAT1, HMGCR, HMGCS1* |
|  | PC3 | hsa04141:Protein processing in endoplasmic reticulum | 6 | 3.95 | 0.00731889 | *PDIA4, HERPUD1, EDEM3, DNAJB11, DNAJC3, HSPA5* |
|  |  | hsa04070:Phosphatidylinositol signaling system | 4 | 2.63 | 0.033926142 | *PI4K2B, PPIP5K2, PIK3CB, MTMR3* |
| **Downregulated genes** | A375 | hsa05016:Huntington's disease | 7 | 6.93 | 0.000493896 | *NDUFS7, POLR2F, CLTB, POLR2I, NDUFA13, NDUFA1, UQCR11* |
|  |  | hsa05010:Alzheimer's disease | 6 | 5.94 | 0.001894793 | *NDUFS7, CDK5R1, NDUFA13, NDUFA1, UQCR11, APBB1* |
|  |  | hsa00190:Oxidative phosphorylation | 5 | 4.95 | 0.005381222 | *ATP5I, NDUFS7, NDUFA13, NDUFA1, UQCR11* |
|  |  | hsa05012:Parkinson's disease | 4 | 3.96 | 0.040147596 | *NDUFS7, NDUFA13, NDUFA1, UQCR11* |
|  |  | hsa04932:Non-alcoholic fatty liver disease (NAFLD) | 4 | 3.96 | 0.046782126 | *NDUFS7, NDUFA13, NDUFA1, UQCR11* |
|  | A549 |  |  |  |  |  |
|  | MCF7 | hsa05145:Toxoplasmosis | 3 | 5.17 | 0.04754484 | *PPIF, BIRC7, GNAO1* |
|  | PC3 |  |  |  |  |  |

Table S4. All merged gene list of GO enrichment analysis

|  | **Biological process (BP)** | **Counts** | **%** | ***p*-value** | **Genes** |
| --- | --- | --- | --- | --- | --- |
| **Upregulated genes** | GO:0006695~cholesterol biosynthetic process | 12 | 22.22 | 1.15E-19 | *SQLE, IDI1, MVK, HMGCS1, INSIG1, MSMO1, DHCR24, HMGCR, DHCR7, HSD17B7, LSS, FDFT1* |
|  | GO:0055114~oxidation-reduction process | 11 | 20.37 | 1.36E-05 | *SQLE, AOC2, SCD, FASN, MSMO1, DHCR24, GLRX, HMGCR, DHCR7, HSD17B7, FDFT1* |
|  | GO:0045454~cell redox homeostasis | 6 | 11.11 | 4.29E-06 | *NME9, KRIT1, TXNDC11, GLRX, TXNDC15, PDIA4* |
|  | GO:0008299~isoprenoid biosynthetic process | 5 | 9.26 | 8.65E-08 | *IDI1, MVK, HMGCS1, HMGCR, FDFT1* |
|  | GO:0036498~IRE1-mediated unfolded protein response | 5 | 9.26 | 3.54E-05 | *DNAJC3, HSPA5, GFPT1, DNAJB11, DNAJB9* |
|  | GO:0008203~cholesterol metabolic process | 5 | 9.26 | 6.20E-05 | *SQLE, INSIG1, APOL1, LDLR, SREBF2* |
|  | GO:0070588~calcium ion transmembrane transport | 5 | 9.26 | 5.37E-04 | *SLC24A2, CACFD1, TRPV2, TRPM7, CACNA2D4* |
|  | GO:0035338~long-chain fatty-acyl-CoA biosynthetic process | 4 | 7.41 | 3.12E-04 | *SCD, FASN, ACSL4, ELOVL6* |
|  | GO:0006629~lipid metabolic process | 4 | 7.41 | 0.013350468 | *HMGCS1, ACSL4, LDLR, SREBF2* |
|  | GO:0051260~protein homooligomerization | 4 | 7.41 | 0.018348168 | *P2RX6, EIF2AK3, PRND, KCTD7* |
|  | GO:0006457~protein folding | 4 | 7.41 | 0.019175978 | *RANBP2, DNAJB11, TXNDC11, PDIA4* |
|  | GO:0001525~angiogenesis | 4 | 7.41 | 0.033299113 | *CLIC4, KRIT1, EIF2AK3, CTGF* |
|  | GO:0010628~positive regulation of gene expression | 4 | 7.41 | 0.049711987 | *EIF2AK3, KRAS, LDLR, CTGF* |
|  | GO:0008033~tRNA processing | 3 | 5.56 | 0.006065588 | *LSM6, TRMT12, PUS7L* |
|  | GO:0051262~protein tetramerization | 3 | 5.56 | 0.0070611 | *NUDT21, TRPM7, HMGCR* |
|  | GO:0007077~mitotic nuclear envelope disassembly | 3 | 5.56 | 0.008495267 | *RANBP2, NUP107, LPIN1* |
|  | GO:0008542~visual learning | 3 | 5.56 | 0.008872553 | *KRAS, HMGCR, MEIS2* |
|  | GO:0034976~response to endoplasmic reticulum stress | 3 | 5.56 | 0.023420192 | *EIF2AK3, TXNDC11, PDIA4* |
|  | GO:0001503~ossification | 3 | 5.56 | 0.026404853 | *EIF2AK3, CTGF, DDR2* |
|  | GO:1902476~chloride transmembrane transport | 3 | 5.56 | 0.034833745 | *CLCN6, CLIC4, APOL1* |
|  | GO:0033490~cholesterol biosynthetic process via lathosterol | 2 | 3.70 | 0.01256653 | *DHCR24, DHCR7* |
|  | GO:0033489~cholesterol biosynthetic process via desmosterol | 2 | 3.70 | 0.01256653 | *DHCR24, DHCR7* |
|  | GO:0051385~response to mineralocorticoid | 2 | 3.70 | 0.01256653 | *KRAS, CTGF* |
|  | GO:0042759~long-chain fatty acid biosynthetic process | 2 | 3.70 | 0.018791562 | *SCD, ELOVL6* |
|  | GO:0070508~cholesterol import | 2 | 3.70 | 0.018791562 | *STARD4, LDLR* |
|  | GO:0070417~cellular response to cold | 2 | 3.70 | 0.021889623 | *DNAJC3, EIF2AK3* |
|  | GO:0035988~chondrocyte proliferation | 2 | 3.70 | 0.02805698 | *CTGF, DDR2* |
|  | GO:2000188~regulation of cholesterol homeostasis | 2 | 3.70 | 0.02805698 | *LDLR, SREBF2* |
|  | GO:0016126~sterol biosynthetic process | 2 | 3.70 | 0.031126335 | *SQLE, MSMO1* |
|  | GO:0006983~ER overload response | 2 | 3.70 | 0.031126335 | *HSPA5, EIF2AK3* |
|  | GO:0036499~PERK-mediated unfolded protein response | 2 | 3.70 | 0.037236542 | *HSPA5, EIF2AK3* |
|  | GO:0008610~lipid biosynthetic process | 2 | 3.70 | 0.043308937 | *ACSL4, FDFT1* |
|  | GO:0006636~unsaturated fatty acid biosynthetic process | 2 | 3.70 | 0.046331027 | *SCD, ELOVL6* |
| **Downregulated genes** | GO:0045944~positive regulation of transcription from RNA polymerase II promoter | 8 | 22.86 | 0.003047762 | *NEUROD2, MED25, CIITA, MYC, E2F4, HOXC13, ELK1, JUNB* |
|  | GO:0008284~positive regulation of cell proliferation | 7 | 20.00 | 3.07E-04 | *CST3, CSF1R, MYC, CLCF1, ZNF703, MATK, EMP2* |
|  | GO:0006366~transcription from RNA polymerase II promoter | 7 | 20.00 | 5.13E-04 | *NEUROD2, MYC, E2F4, HOXC13, ZBTB7B, ELK1, JUNB* |
|  | GO:0030154~cell differentiation | 5 | 14.29 | 0.013656629 | *MATK, SPATA31D1, ZBTB7B, ELK1, GTSF1* |
|  | GO:0042493~response to drug | 4 | 11.43 | 0.023242148 | *GNAO1, CST3, MYC, JUNB* |
|  | GO:0008283~cell proliferation | 4 | 11.43 | 0.037339319 | *CSF1R, MYC, MATK, EMP2* |
|  | GO:0030316~osteoclast differentiation | 3 | 8.57 | 9.80E-04 | *CSF1R, GPR183, JUNB* |
|  | GO:0001541~ovarian follicle development | 3 | 8.57 | 0.003256718 | *LFNG, MYC, FANCG* |
|  | GO:0006120~mitochondrial electron transport, NADH to ubiquinone | 3 | 8.57 | 0.004408983 | *NDUFA13, NDUFS7, NDUFA1* |
|  | GO:0071277~cellular response to calcium ion | 3 | 8.57 | 0.004768075 | *NEUROD2, PPIF, JUNB* |
|  | GO:0032981~mitochondrial respiratory chain complex I assembly | 3 | 8.57 | 0.00719368 | *NDUFA13, NDUFS7, NDUFA1* |
|  | GO:0019221~cytokine-mediated signaling pathway | 3 | 8.57 | 0.028794175 | *CSF1R, CLCF1, IL20RA* |
|  | GO:0018108~peptidyl-tyrosine phosphorylation | 3 | *8.57* | 0.038254241 | *CSF1R, ZMYM2, FGFR1OP2* |
|  | GO:0045348~positive regulation of MHC class II biosynthetic process | 2 | *5.71* | 0.016087191 | *CIITA, AZU1* |
|  | GO:0045124~regulation of bone resorption | 2 | *5.71* | 0.018080341 | *CSF1R, IL20RA* |
|  | GO:0043534~blood vessel endothelial cell migration | 2 | *5.71* | 0.020069573 | *GPX1, EMP2* |
|  | GO:0006884~cell volume homeostasis | 2 | *5.71* | 0.020069573 | *E2F4, KCNN4* |
|  | GO:0071257~cellular response to electrical stimulus | 2 | *5.71* | 0.024036308 | *NEUROD2, CIITA* |
|  | GO:0090201~negative regulation of release of cytochrome c from mitochondria | 2 | *5.71* | 0.033885083 | *GPX1, PPIF* |
|  | GO:2001243~negative regulation of intrinsic apoptotic signaling pathway | 2 | *5.71* | 0.043637366 | *NDUFA13, PPIF* |

Table S5. All merged gene list of KEGG pathway enrichment analysis

|  | **Pathway** | **Counts** | **%** | ***p*-value** | **Genes** |
| --- | --- | --- | --- | --- | --- |
| **Upregulated genes** | hsa01100:Metabolic pathways | 16 | 29.63 | 3.11E-04 | *IDI1, MVK, AOC2, HMGCS1, GFPT1, MSMO1, ACSL4, DHCR24, HMGCR, HSD17B7, LSS, SQLE, FASN, DHCR7, LPIN1, FDFT1* |
|  | hsa01130:Biosynthesis of antibiotics | 10 | 18.52 | 5.68E-07 | *SQLE, IDI1, MVK, HMGCS1, GFPT1, MSMO1, HMGCR, HSD17B7, LSS, FDFT1* |
|  | hsa00100:Steroid biosynthesis | 7 | 12.96 | 3.38E-10 | *SQLE, MSMO1, DHCR24, DHCR7, HSD17B7, LSS, FDFT1* |
|  | hsa04141:Protein processing in endoplasmic reticulum | 5 | 9.26 | 0.009177235 | *DNAJC3, HSPA5, DNAJB11, EIF2AK3, PDIA4* |
|  | hsa00900:Terpenoid backbone biosynthesis | 4 | 7.41 | 1.59E-04 | *IDI1, MVK, HMGCS1, HMGCR* |
|  | hsa01212:Fatty acid metabolism | 4 | 7.41 | 0.001639359 | *SCD, FASN, ACSL4, ELOVL6* |
| **Downregulated genes** | hsa05012:Parkinson's disease | 4 | 11.43 | 0.00872218 | *NDUFA13, NDUFS7, NDUFA1, PPIF* |
|  | hsa05016:Huntington's disease | 4 | 11.43 | 0.019659352 | *NDUFA13, NDUFS7, NDUFA1, PPIF* |
|  | hsa05145:Toxoplasmosis | 3 | 8.57 | 0.043668972 | *GNAO1, CIITA, PPIF* |

Table S6. GO enrichment analysis results of potential targets.

| **Term** | **Count** | **%** | ***p*-value** | **Genes** |
| --- | --- | --- | --- | --- |
| GO:0042493~response to drug | 5 | 33.3 | 9.13E-05 | *HTR2A, DRD2, SLC6A2, ADRA1A, SLC6A4* |
| GO:0007613~memory | 4 | 26.7 | 1.69E-05 | *HRH2, HTR2A, PLA2G6, SLC6A4* |
| GO:0051967~negative regulation of synaptic transmission glutamatergic | 3 | 20 | 2.32E-05 | *HTR2A, DRD2, PLA2G6* |
| GO:0045907~positive regulation of vasoconstriction | 3 | 20 | 3.16E-04 | *HRH2, HTR2A, ADRA1A* |
| GO:0008542~visual learning | 3 | 20 | 6.26E-04 | *HRH2, DRD2, MTOR* |
| GO:0016049~cell growth | 3 | 20 | 9.69E-04 | *AR, ADRA1A, MTOR* |
| GO:0007204~positive regulation of cytosolic calcium ion concentration | 3 | 20 | 0.005401637 | *CACNA1C, PLA2G6, ADRA1A* |
| GO:0070374~positive regulation of ERK1 and ERK2 cascade | 3 | 20 | 0.009050879 | *HTR2A, DRD2, ADRA1A* |
| GO:0007268~chemical synaptic transmission | 3 | 20 | 0.016531193 | *HTR2A, SLC6A2, SLC6A4* |
| GO:0010628~positive regulation of gene expression | 3 | 20 | 0.019503036 | *AR, MTOR, SLC6A4* |
| GO:0006810~transport | 3 | 20 | 0.033064439 | *AR, CACNA1I, SLC6A2* |
| GO:0008285~negative regulation of cell proliferation | 3 | 20 | 0.041867244 | *AR, DRD2, ADRA1A* |
| GO:0035556~intracellular signal transduction | 3 | 20 | 0.043218986 | *PLCL1, DRD2, ADRA1A* |
| GO:0014832~urinary bladder smooth muscle contraction | 2 | 13.3 | 0.00333105 | *HTR2A, PLA2G6* |
| GO:0090037~positive regulation of protein kinase C signaling | 2 | 13.3 | 0.006651795 | *PLA2G6, ADRA1A* |
| GO:0015844~monoamine transport | 2 | 13.3 | 0.006651795 | *SLC6A2, SLC6A4* |
| GO:0045945~positive regulation of transcription from RNA polymerase III promoter | 2 | 13.3 | 0.009135608 | *AR, MTOR* |
| GO:0030431~sleep | 2 | 13.3 | 0.009135608 | *CACNA1I, HTR2A* |
| GO:0032228~regulation of synaptic transmission, GABAergic | 2 | 13.3 | 0.009962263 | *PLCL1, DRD2* |
| GO:0014059~regulation of dopamine secretion | 2 | 13.3 | 0.010788278 | *HTR2A, DRD2* |
| GO:0003382~epithelial cell morphogenesis | 2 | 13.3 | 0.011613653 | *AR, HRH2* |
| GO:0060402~calcium ion transport into cytosol | 2 | 13.3 | 0.011613653 | *CACNA1C, ADRA1A* |
| GO:0048148~behavioral response to cocaine | 2 | 13.3 | 0.011613653 | *HTR2A, DRD2* |
| GO:0006939~smooth muscle contraction | 2 | 13.3 | 0.014908761 | *HTR2A, ADRA1A* |
| GO:0001659~temperature homeostasis | 2 | 13.3 | 0.015730943 | *HTR2A, DRD2* |
| GO:0071880~adenylate cyclase-activating adrenergic receptor signaling pathway | 2 | 13.3 | 0.015730943 | *DRD2, ADRA1A* |
| GO:0043278~response to morphine | 2 | 13.3 | 0.019013302 | *DRD2, MTOR* |
| GO:0050482~arachidonic acid secretion | 2 | 13.3 | 0.019832302 | *PLA2G4A, DRD2* |
| GO:0036152~phosphatidylethanolamine acyl-chain remodeling | 2 | 13.3 | 0.019832302 | *PLA2G4A, PLA2G6* |
| GO:0060135~maternal process involved in female pregnancy | 2 | 13.3 | 0.020650668 | *PLA2G6, MTOR* |
| GO:0007202~activation of phospholipase C activity | 2 | 13.3 | 0.021468398 | *HTR2A, ADRA1A* |
| GO:0036151~phosphatidylcholine acyl-chain remodeling | 2 | 13.3 | 0.022285495 | *PLA2G4A, PLA2G6* |
| GO:0007616~long-term memory | 2 | 13.3 | 0.023101958 | *DRD2, MTOR* |
| GO:0042220~response to cocaine | 2 | 13.3 | 0.026361483 | *DRD2, MTOR* |
| GO:0051209~release of sequestered calcium ion into cytosol | 2 | 13.3 | 0.033658526 | *HTR2A, DRD2* |
| GO:0035176~social behavior | 2 | 13.3 | 0.03929886 | *MTOR, SLC6A4* |
| GO:0051259~protein oligomerization | 2 | 13.3 | 0.047303466 | *AR, SLC6A4* |

Table S7. The results of KEGG pathway enrichment analysis of potential targets.

| **Term** | **Count** | **%** | ***p*-value** | **Genes** |
| --- | --- | --- | --- | --- |
| hsa04020:Calcium signaling pathway | 5 | 33.3 | 1.86E-04 | *CACNA1I, HRH2, CACNA1C, HTR2A, ADRA1A* |
| hsa04726:Serotonergic synapse | 4 | 26.7 | 8.09E-04 | *PLA2G4A, CACNA1C, HTR2A, SLC6A4* |
| hsa04270:Vascular smooth muscle contraction | 4 | 26.7 | 9.43E-04 | *PLA2G4A, CACNA1C, PLA2G6, ADRA1A* |
| hsa04080:Neuroactive ligand-receptor interaction | 4 | 26. 7 | 0.010842037 | *HRH2, HTR2A, DRD2, ADRA1A* |
| hsa04750:Inflammatory mediator regulation of TRP channels | 3 | 20 | 0.012082169 | *PLA2G4A, HTR2A, PLA2G6* |
| hsa04728:Dopaminergic synapse | 3 | 20 | 0.02006995 | *CALY, CACNA1C, DRD2* |
| hsa00592:alpha-Linolenic acid metabolism | 2 | 13.3 | 0.042783288 | *PLA2G4A, PLA2G6* |
| hsa00591:Linoleic acid metabolism | 2 | 13.3 | 0.049470753 | *PLA2G4A, PLA2G6* |
